# Oxidized Cell-Free DNA Is a Factor of Stress Signaling in Radiation-Induced Bystander Effects in Different Types of Human Cells

**DOI:** 10.1155/2019/9467029

**Published:** 2019-08-19

**Authors:** Marina S. Konkova, Andrew A. Kaliyanov, Vasilina A. Sergeeva, Margarita S. Abramova, Svetlana V. Kostyuk

**Affiliations:** Research Centre for Medical Genetics (RCMG), Moscow 115478, Russia

## Abstract

In pathology or under damaging conditions, the properties of cell-free DNA (cfDNA) change. An example of such change is GC enrichment, which drastically alters the biological properties of cfDNA. GC-rich cfDNA is a factor of stress signaling, whereas genomic cfDNA is biologically inactive. GC-rich cfDNA stimulates TLR9-MyD88-NF-*κ*B signaling cascade, leading to an increase in proinflammatory cytokine levels in the organism. In addition, GC-rich DNA is prone to oxidation and oxidized cfDNA can stimulate secondary oxidative stress. This article is a review of works dedicated to the investigation of a low-dose ionizing radiation effect, a bystander effect, and the role of cfDNA in both of these processes.

## 1. Circulating Cell-Free DNA

The presence of DNA in the noncellular fraction of peripheral blood (cell-free DNA) was initially identified more than 50 years ago [1]. cfDNA was shown to be present in the blood of healthy subjects. The main source of cfDNA is necrotically and apoptotically dying cells [2]. Some authors suggest that cfDNA can be synthesized and actively excreted to the medium by stimulated cells [3, 4]. For a long time, cfDNA was studied as a passive marker of cell death in many diseases and conditions such as cancer, autoimmune and cardiovascular diseases, and various types of stress, including radiation-induced [5]. cfDNA is an object for noninvasive diagnostics (liquid biopsy), including prenatal diagnostics.

Increased interest in cfDNA is associated with the possibility of its use for diagnostic purposes. Tumor cfDNA can be used for early diagnosis, monitoring, and therapeutic prognosis of different types of cancer, including the analysis of the genome of tumor cells as well as for noninvasive detection of pregnancy pathology and disorders of fetal development [6]. cfDNA can also be used to assess the risk of damaging factors, including ionizing radiation and ultraviolet radiation [7, 8]. cfDNA is used as a marker of pathology in autoimmune diseases, in acute conditions that lead to death of a large number of cells (stroke, myocardial infarction), sepsis, transplantation, and trauma [9, 10]. The latest data suggests that serum also contains circulating RNA, which can serve as a marker of many pathologies [11].

In various pathological conditions and under stress conditions, the concentration of cfDNA tends to increase sharply. However, in some cases, despite the high level of cell death in the organism, the concentration of cfDNA in the bloodstream can appear significantly decreased due to endogenous or exogenous reasons [12]. The analysis of cfDNA of people working under conditions of increased radiation background (regularly exposed to low doses of gamma-neutron radiation or radiation of tritium) revealed the following. Instead of the expected increase in cfDNA concentration due to increased apoptosis under the action of reactive oxygen species (ROS), a significant decrease of cfDNA concentration was observed compared to a control sample of nonirradiated healthy donors living in the same area [12]. Despite this, comet assay data indicated elevated levels of DNA breaks in the lymphocytes of these subjects. The study revealed that irradiation increases the activity of the main enzyme responsible for the hydrolysis of DNA, namely, DNase I [12]. Thus, the decrease in the concentration of cfDNA when the cell death level is high can be explained with the elimination of cfDNA from the bloodstream.

It is a well-known fact that circulating cell-free DNA contains a higher percentage of GC pairs than genomic DNA [13]. Under chronic oxidative stress conditions, cfDNA accumulates GC-rich sequences of the genome. The human genome contains three major GC-rich moderate repeats in almost equal parts: circular mitochondrial DNA (mtDNA), telomere repeat (telDNA), and the transcribed region of tandem ribosomal repeat (TR-rDNA) coding for major ribosomal RNA. All the three repeats are accumulated in the total pool of human cfDNA with time, i.e., their fraction trends to increase with time. Much attention is paid by most authors to the immunomodulatory action of extracellular mtDNA [14, 15]. The proportion of mitochondrial DNA in cfDNA is increased under conditions of oxidative stress, and since mitochondrial DNA contains large amounts of 8-oxodG compared to the genomic DNA, the pool of cfDNA becomes enriched with oxidized fragments. To a lesser degree, the extracellular telDNA [16] and ribosomal repeat within cfDNA [17] are explored.

This DNA is, firstly, enriched with GC-rich motifs, including unmethylated CpG motifs, which are recognized by TLR9 receptors and stimulate TLR9-MyD88-NF-kB signaling cascade, activation of which leads to an increase in the concentration of proinflammatory cytokines in the organism. Secondly, this DNA contains a large count of oxidized and/or easily oxidizable (dG)n fragments. These two new characteristics make cfDNA a biologically active molecule [10, 18].

Also, a number of recently released publications discuss changes in cfDNA properties that turn cfDNA in a biologically active molecule. cfDNA may enhance oxidative stress, stimulate the synthesis of proinflammatory cytokines, and induce sterile inflammation, and it is able to activate a large number of signaling pathways in various cell types [19]. It was shown that while both cfDNA of cancer patients and of healthy donors can induce apoptosis in vitro and in vivo, cfDNA of healthy donors has a less prominent detrimental effect. Simultaneous treatment with cfDNA and DNase I eliminates the damaging effects of cfDNA [20]. It was shown that cfDNA can stimulate immune response, affect cell proliferation, and inhibit induced secretion of proinflammatory cytokines [19]. An idea that cfDNA fragments can be integrated into the genome of normal cells, causing their transformation, forms the genometastasis hypothesis [21]. According to some authors, metastasis occurs through transfection of susceptible cells in distant organs via the dominant oncogenes that circulate in the plasma in the composition of cfDNA [22].

Thus, it has become apparent that activation of the immune system may occur not only due to foreign stimuli, such as bacteria and viruses, but also under the influence of endogenous biomolecules that appear in bloodstream as a result of organism cell death. Pool of these biomolecules is collectively known as damage-associated molecular patterns (DAMPs) [23]. DAMPs cause changes in the functional activity of healthy cells of the organism, inducing the synthesis of pro- and anti-inflammatory cytokines, sterile inflammation, and adaptive response. Proteins (especially the protein HMGB1), lipoproteins, and their oxidation products are the most researched DAMPs. Сirculating cfDNA was not considered as a biologically active component of the pool of DAMPs until recently, as it has been previously shown that the DNA of mammalian genomes has a weak immune stimulating effect. However, recent studies have shown that cfDNA is a stress signaling DAMP [3, 10, 19]. cfDNA affects many parameters of the organism. Circulating cfDNA can increase oxidative stress, stimulate the synthesis of proinflammatory cytokines, induce sterile inflammation, affect platelet activation, plasmatic coagulation, and fibrinolysis, and activate a large number of signaling pathways [24, 25].

## 2. Low-Dose Ionizing Radiation (LDIR)

The study of the biological effects of low-dose radiation remains in the focus of scientific research due to the inevitable exposure of human cells to ionizing radiation. People are commonly exposed to low-dose ionizing radiations (LDIR) over natural background levels (terrestrial and cosmic), may be exposed for medical diagnostics or accidentally (illegal radioactive waste dumpsites, nuclear accidents). An average person is exposed to small doses of ionizing radiation much more frequently than to high-dose radiation.

During the early decades of the 20th century, the consensus of the public had been achieved that the most fundamental radiation dose-response relationships have a threshold [26]. However, subsequently, the dose-response model was replaced with a conservative model with the linear no-threshold (LNT) hypothesis that there is no threshold to induce radiation response [27] according to which even the smallest doses of IR could potentially increase the cancer risk.

Investigation of the effect of LDIR on the cells revealed phenomena that do not fit the traditional idea of direct DNA damage by radiation. Experimental and epidemiological evidences show that the LNT model is not appropriate for damage assessment, including calculation of cancer risks at low doses [28]. The effect of LDIR on the expression of genes controlling apoptosis, cell cycle progression, proliferation, and differentiation was investigated in a number of studies [29, 30]. It was reported that exposure to IR at doses above 0.05-0.1 Gy (protracted exposure) or 0.01-0.05 Gy (acute exposure) increases the risk of some cancers [31]. But a number of epidemiological studies are available for LDIR exposures below 0.1 Gy on stochastic effects such as cancer incidence and effects on heredity [32], and it was reported that 0.06 Gy of LDIR exposure might increase the risk of brain cancer threefold [33]. In addition, it was discovered that radiation can cause chromosomal instability in cells not directly affected by radiation tracks (bystander effect). LDIR can also lead to the development of the adaptive response and hormesis, which is of importance when it comes to public health issues [28].

## 3. Oxidative Stress

LDIR can have a direct effect when quantums of energy affect the cell structure and through the radiolysis of water with formation of free radicals and other highly reactive substances. The disruption of the balance between the formation of free radicals and the antioxidant defense system activity is called oxidative stress. In conditions of oxidative stress caused by external impact (for example, under the action of LDIR), or in chronic pathology, the level of oxidative modification of nuclear DNA is significantly increased [34]. Cells with a high level of unrepaired oxidative DNA damage die, adding oxidized DNA fragments to the pool of cfDNA [10]. All DNA bases can be oxidized to varying degrees [34]. The main products of oxidation of nuclear DNA are thymidine glycol and 8-hydroxy-2′-deoxyguanosine (8-oxodG) [10, 35]. The most widely used “marker” for oxidative DNA damage is 8-oxodG. 8-OxodG is formed in DNA either through direct oxidation of nucleic acids or may be incorporated from nucleotide pool by the DNA polymerase [35]. It has been shown that mitochondrial DNA is the main carrier of oxidized DNA bases [36]. The content of 8-oxodG is about 20 bases per million nucleotides in intact mitochondrial DNA and about 300 bases per million of nucleotides in mitochondrial DNA under oxidative stress [36]. Particularly high contents of 8-охоdG within cfDNA are observed in cancer patients and patients with cardiovascular diseases, where it can reach 3000 8-oxodG per million nucleotides [37].

## 4. Bystander Effect

RIBE refers to the nontargeted effects (NTE)—the effects in cells or organisms that are not exposed to the direct damaging effect of ionizing radiation [38]. RIBE consists in a specific response of nonirradiated cells to molecular signals sent by cells exposed to the LDIR. RIBE is described for cells, tissues, and whole organisms and is believed to be linked to the mechanisms coordinating the response at higher levels of the organization [39]. These have been shown to predominate in the low-dose region of the dose response curve and can be seen at doses below the threshold for adverse effects, such as mutation or impacts on mortality or morbidity [40]. For X-ray, RIBE can be observed starting with doses of 5 mGy, where the level of DNA damage of target cells is only five single-strand DNA breaks, 10-15 damaged bases, and one double-strand break in every fifth cell. This effect is observed even when a single cell is irradiated with only one *α*-particle and appears in the range of low doses from 1 to 50 cGy [41]. Manifestations of RIBE are often seen as double-strand DNA breaks (DSB), genomic instability, and reduced cell viability [42]. In some cases, RIBE leads to the development of the adaptive response (AR), which gives the bystander cells resistance to damaging radiation doses. AR and RIBE have a lot in common and are closely interconnected [43]. The type of cellular response depends on the radiation dose, cell type, and stage of the cell cycle.

The existence of signaling molecules informing the unexposed cells or organisms leaves no doubt, but their nature causes heated discussions [39]. The signal transmission from irradiated cells to “bystander” cells may occur in one of two ways or their combination: the first is by gap-junction intercellular communication (GJIC) [44] and the second is by extracellular soluble factors [45]. Both ways were discovered around the same time by separate researchers. It was subsequently discovered that the physical signals may also play a role and that the UV photons from irradiated cells can also generate responses of “bystander” cells [46].

Both GJIC and extracellular soluble factor mechanisms were eventually confirmed to independently lead to similar effects [44, 47]. Perhaps later on, a “unifying mechanism” will be found. The search for the signal molecule that can induce RIBE is ongoing. A lot of soluble extracellular factors were suggested as RIBE signals over the years, in particular cytokines, including IL-6 and IL-8 [48], transforming growth factor-b1 (TGFb1) [49] and TNFa, NO, and various types of reactive oxygen species (ROS) [50]. There is also the possibility that RIBE may be a manifestation of a generalized response to cellular stress [51]. However, it is highly likely that this signaling molecule (stress signal) is not just a specific molecule but rather a complex effect in which every component plays a distinct role and function. It is probable that cytokines transmit the signal over long distances, whereas short distance signals are conducted by unstable but highly reactive ROS or locally released NO. On the other hand, the signal molecule may be a soluble factor that functions in various types of cells both locally and at a considerable distance. This factor may occur as a result of apoptotic or necrotic cell death, as well as death of cancerous or senescent cells. This factor must be able to enter the bloodstream and circulate there for a considerable period of time, providing system-wide stimulation of the relevant signaling pathways. It is also likely that this “universal factor” described above activates the synthesis of compounds normally considered as a stress signal in RIBE—cytokines or ROS.

## 5. Oxidized Cell-Free DNA Is a Factor of Stress Signaling

It is possible that cfDNA can serve as such universal stress signal. Oxidized cfDNA fragments have the most pronounced properties of signaling molecules [52, 53].

As mentioned above, exposure to LDIR leads to an increase in the ROS level within a few seconds to 2-5 minutes [10], resulting in oxidative stress and significantly increased level of oxidative modification of DNA. Cells with high levels of oxidative damage die due to apoptosis or necrosis, and the released cfDNA fragments are oxidized and enriched with GC bases compared to the normal nuclear DNA [10]. GC-rich fragments of DNA are prone to oxidation under these conditions which adds oxidized cfDNA to the cfDNA pool. Chronic exposure to gamma-neutron radiation or radiation of tritium causes an increase in the amount of GC-rich sequences (69% GC) of the transcribed region of the human ribosomal repeat (TR-rDNA) in cfDNA from 166 individuals. The reason for this phenomenon is the increased stability of GC repeats towards hydrolysis [17]. The transcribed TR-rDNA region is one of the examples of preferred oxidation of DNA. We have shown that cfDNA from irradiated cells contains a significantly larger amount of 8-oxodG than cfDNA from control (unirradiated) cells or cellular DNA of irradiated cells [52].

The role of cfDNA extracted from the dying irradiated cells as a mediator of RIBE was studied on various types of human cells: G0 lymphocytes of peripheral blood [10, 54], endothelial cell umbilical vein (HUVEC) [55], and mesenchymal stem cells (MSC) [56]. The typical design of the experiment included low-dose irradiation of cells (10 cGy) with subsequent culturing for 1-3 h followed by removal of the cells and extraction of cfDNA from the media.

cfDNA fragments extracted from the environment of the irradiated cells (cfDNA^R^) and unirradiated (control) cells were added to the medium of nonirradiated (bystander) cells, followed by a detailed analysis. cfDNA^R^ induces an increase in ROS production approximately to the same extent as LDIR [54, 55], whereas the control cfDNA does not induce the synthesis of ROS. It should be noted that the increase in ROS production is a very common type of reaction to stress and is found after irradiation and after adding cfDNA^R^ in the culture medium [53, 54]. In both irradiated and bystander cells, the increase in ROS levels is accompanied by an increase in the number of double-stranded DNA breaks (DSB) [53]. In turn, the presence of a DSB induces a DNA Damage Response (DDR), which involves a change in the structure of chromatin and transfer of chromatin regions in the nucleus. Rearrangement of chromatin was observed in response to irradiation (3-50 cGy) or impact cfDNA^R^ in G0 lymphocytes and HUVEC and MSC. FISH revealed the migration of pericentromeric loci of chromosome 1 (1q12) relative to the center of the nucleus and each other [10, 56]. All of these effects were mainly dependent on the increase of ROS production and were blocked when ROS scavenger *α*-tocopherol was added to the medium. Changes in the spatial structures of chromatin require dynamic transformation of the cytoskeleton, which is achieved in the process of polymerization/depolymerization of actin [57]. It is known that ROS affect the rate of polymerization of actin and the motility and adhesion of cultured cells [58]. cfDNA^R^ stimulates the formation of F-actin in unirradiated HUVEC cells; the effect similar to that is observed in response to LDIR. cfDNA from control cell does not cause activation of actin polymerization [55]. It seems that the events of the chromatin remodeling that are observed after irradiation or exposure to cfDNA^R^ are interlinked and subordinated to the overall goal of ensuring the transcription or silencing of genes that have to change their activity in response to the impact. cfDNA^R^ extracted from the medium of irradiated cells causes a decrease in the number of cells with DNA breaks in intact endothelial cells; the effect similar to that was observed when cells were irradiated in small doses. The incubation medium of irradiated cells induces the initial stage of the apoptotic cascade in bystander cells, which are accompanied by an increase in the content of ROS within 6 hours [47]. These examples show that the effects caused by LDIR can be transmitted through the culture medium (or extracellular space in the body), and cfDNA is the most likely signal molecule in RIBE. Further proof of this hypothesis is the fact that cfDNA from nonirradiated cells does not lead to the above effects, and no adaptive response is observed. Moreover, if the cfDNA from the irradiated cells medium is treated with DNase I, it loses the ability to induce adaptive response.

In addition to increased levels of ROS development, cfDNA^R^-dependent RIBE requires apoptosis of a part of the irradiated cells. The dying cells are a major source cfDNA^R^. Inhibition of caspase-3 in irradiated G0 lymphocytes that is known to block apoptotic cascade results in loss of the signal properties cfDNA^R^ [53].

The assumption is that oxidized cfDNA fragments penetrate into the cells, as was shown previously [52], and the key factor is the oxidation of the DNA. A genetic construction containing a (G)n repetition that is prone to oxidation was used to show that oxidized cfDNA can quickly penetrate into the cytoplasm and stimulate short-term increase in the production of ROS, caused by NOX4 oxidase. This leads to transient oxidative modification of nuclear DNA but also activates the antioxidant system. Elevated levels of ROS lead to DNA damage and DSB, but at the same time, activates DNA repair and levels the damage. In addition, LDIR (10 cGy) induces a strong antiapoptotic response.

The secondary oxidative stress in intact bystander cells is caused by the interaction of cfDNA^R^ with the receptor/DNA sensors that are present either on the surface or inside the bystander cells. The toll-like receptors are among the possible candidates for such sensors [59]. In the populations of irradiated lymphocytes, the levels of both TLR9 and its main adaptor MyD88 increase several times [53]. Oxidized DNA and GC-rich fragments of the cfDNA are stronger TLR9-stimulating ligands than nonoxidized DNA fragments [54, 55]. After the formation of the complex, DNA-TLR9 signaling in the downstream direction activates the transcription factor NF-kB, which increases the biosynthesis of ROS in various ways [54, 59]. Blocking TLR9 levels some of the observed effects [54, 55]. For example, when the TLR9 pathway is blocked in irradiated G0 lymphocytes, there is no significant change in the localization of loci 1q12 or in the level of ROS [53]. Evidence pointing at the existence of the toll-like receptor-independent stress signal transfer pathways was demonstrated by other authors, including cytoplasmic DNA-dependent STING, AIM2, RIG-1, and DAI sensor pathways [60]. The reception of cfDNA^OX^ produced by irradiated cells requires further investigations.

**Figure 1:**
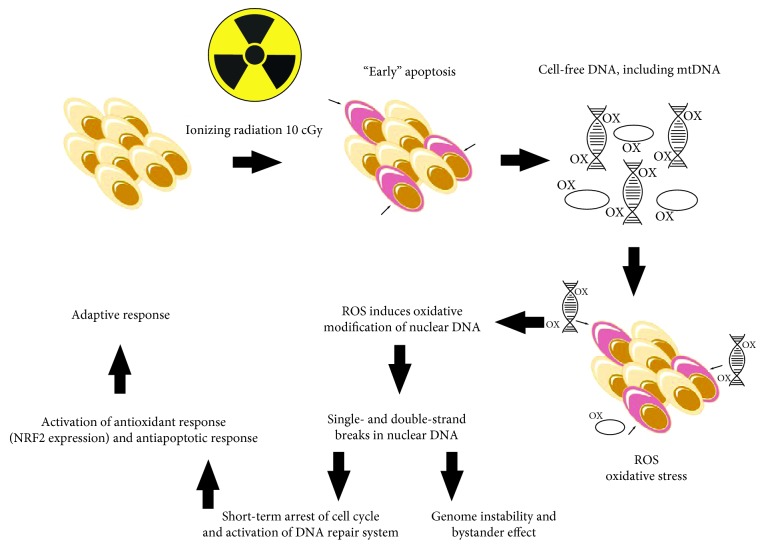
Proposed mechanisms for the development of radioadaptive responses and bystander effect.

The level of ROS increases dramatically during the first minutes after addition of cfDNA^OX^ or cfDNA^R^ to the culture medium of intact cells but reduces 30 min after addition. After 1 h of incubation, cells have moderately elevated the levels of ROS. MSC respond to the oxidation of DNA faster than differentiated cells [56]. Oxidative stress is a key step, which on the one hand, triggers the oxidation and DNA damage in cells but on the other hand, contributes to the development of the adaptive response (activation of DNA reparation, activation of the antioxidant transcription factor NRF2, and inhibition of apoptosis) (Figure 1). We have shown that cfDNA^R^ that appears after irradiation is responsible for stress signaling, which mediates the RIBE and, in addition, is an important component of the development of the RAR for LDIR.
